# Erythroid variant evolving from chronic myeloid leukemia resistant to multiple tyrosine kinase inhibitors: a rare case report

**DOI:** 10.1186/s13000-024-01446-9

**Published:** 2024-01-24

**Authors:** Michelle Hyunju Lee, Amy Song, Julie Y. Li

**Affiliations:** 1 Department of Medical Oncology, Massachusetts General Hospital, 55 Fruit Street, Boston, MA 02114 USA; 2 Department of Internal Medicine, Columbia University Irving Medical Center, 622 W 168th Street, New York, NY 100032 USA; 3 Department of Hematopathology and Lab Medicines, H. Lee Moffitt Cancer Center & Research Institute, 12902 USF Magnolia Drive, Tampa, FL 33612 USA

**Keywords:** Chronic myeloid leukemia, Erythroid variant, Tyrosine kinase inhibitors

## Abstract

**Background:**

Chronic myeloid leukemia (CML) is characterized by the presence of *BCR::ABL1* fusion gene resulting from a reciprocal translocation, t(9;22)(q34;q11.2), leading to prominent granulocytic proliferation. The majority of patients initially present in chronic phase (CP), which may progress to advanced CML with predominantly granulocytic phenotypes in the absence of proper treatment or response to tyrosine kinase inhibitors (TKIs). We present an exceptionally rare case in which an erythroid variant emerged from a CML patient resistant to multiple TKIs. This variant is characterized by the detection of t(9;22) *BCR::ABL1* fusion in erythroid precursors at various maturation stages and the absence of granulocytic progenitor hyperplasia typically seen in classical CML.

**Case presentation:**

A 33-year-old female with CP-CML had received multiple TKI therapies since her initial diagnosis in 2015. Due to intolerable side effects and inconsistent adherence, she exhibited an inadequate response and developed new-onset pancytopenia. Bone marrow (BM) biopsy specimen revealed a hypercellular marrow with significant erythroid hyperplasia (90% of marrow cellularity) and a reversed myeloid-to-erythroid (M: E) ratio of 1:10. Both erythroid and myeloid cells displayed progressive maturation without dysplasia or excess blasts. Chromosomal analysis identified t(9;22) (q34;q11.2) in 19 out of 20 metaphase cells. *BCR::ABL1* fusion transcript (p210 isoform) was confirmed by real-time quantitative polymerase chain reaction (RT-qPCR) and next-generation sequencing (NGS). Notably, no additional pathogenic cytogenetic abnormalities or *ABL1* kinase domain mutations were detected. Here, we report the first published case of an erythroid variant emerging in a CML patient resistant to multiple TKIs—a distinct entity from the erythroid blast crisis evolving from CML.

**Conclusion:**

The erythroid variant of CML is distinguished by the presence of t(9;22) (q34;q11.2) *BCR::ABL1* in predominant erythroid precursors at different stages of maturation. In a myeloid neoplasm showing predominant erythroid hyperplasia without typical CML features, it is vital to correlate morphology and t(9;22) *BCR::ABL1* cytogenetic testing for accurate diagnosis, and to prevent confusion with PEL transformation in CML.

## Background

CML originates from a pluripotent hematopoietic stem cell and is defined by the Philadelphia (Ph) chromosome with t(9;22) (q34.1;q11.2) reciprocal translocation [1]. This abnormality leads to constitutive tyrosine kinase activity, triggering uncontrolled growth in various cell lineages [2]. Patients with CML typically present with leukocytosis with left-shifted granulocytosis in the peripheral blood (PB), and a hypercellular bone marrow (BM) characterized by significant granulocytic proliferation and markedly reduced erythroid cells, resulting in a marked increased M:E ratio > 10:1. It is highly unusual for a CML patient to exhibit predominant erythroid cells in the BM. An erythroid variant of CML has only been reported once, in which the Ph chromosome was detected in different maturing stages of hyperplastic erythroid precursors [3].

Most CML patients are diagnosed in the chronic phase (CP), and without treatment, the disease can progress to the advanced stages: accelerated phase (AP) and/or blast phase (BP) [4]. Tyrosine kinase inhibitors (TKIs) are the standard therapy for CML, significantly improving survival rates and reducing the incidence of progression to advanced phases [5].

The response to TKI therapy is monitored by cytogenetic response (CyR), analyzing the Ph chromosome on conventional bone BM metaphase analysis. Additionally, molecular response (MR) is assessed by measuring *BCR::ABL1* transcription levels using real-time quantitative PCR (RT- qPCR) [6]. The International Scale (IS) serves as the gold standard for quantifying disease burden, expressed as a percentage of *BCR::ABL1* transcripts to an internal control gene (*ABL1*) [7]. MR is expressed as log-reduction from 100%, with major MR defined as a ≥ 3-log reduction (*BCR::ABL1* IS ≤ 0.1%), and deep MR defined as > 4 or 4.5 log reduction (MR4, *BCR::ABL1* IS ≤ 0.01%; or MR4.5, *BCR::ABL1* IS ≤ 0.0032%) [8]. Despite the efficacy of TKI therapy in many cases, some patients develop *BCR::ABL1* kinase domain mutations, leading to treatment resistance [9]. Additionally, some patients develop intolerable side effects that necessitate change in treatment or impact adherence that may increase risk of disease progression [10].

We present a case of a 33-year-old female with CP-CML resistant to multiple TKIs due to suboptimal adherence in the setting of treatment-related toxicities. Despite the absence of cytogenetic progression or *ABL1* kinase mutations, our patient’s CML transformed into a rare erythroid variant, characterized by maturing erythroid precursors harboring t(9;22) *BCR::ABL1.* To the best of our knowledge, this is the first report of an erythroid variant transformation of CML in a patient undergoing TKI treatment.

## Case presentation

In 2015, a previously healthy 26-year-old woman presented with general malaise and headaches. Laboratory evaluation was notable for a total white blood cell (WBC) count of 121.2 × 10^9^/L (reference range (RR) 4.0–11.0 × 10^9^/L) with 3% basophils, 4% eosinophils and 1% circulating myeloblasts. The hemoglobin (Hgb) level was 11.9 g/dL (RR 11.4–15.0 g/dL). An abdominal ultrasound showed a mildly enlarged spleen measuring 14 cm in craniocaudal length, with the EUTOS long-term survival (ETLS) score of 0.86 indicative of low risk. BM biopsy specimen showed a 100% cellular marrow with marked myeloid hyperplasia (myeloid: erythroid (M: E) ratio of 20:1), eosinophilia (10%), basophilia (3%), and < 5% myeloblasts. Both chromosomal and fluorescence in situ hybridization analysis revealed the presence of Ph chromosome in 94% of cells. Chromosomal analysis also identified a common chromosomal polymorphism, inv9 (p12q13), present in all metaphase cells without clinical significance.RT-qPCR for the *BCR::ABL1* fusion transcript p210 was 158.015%. Based on these findings, the patient was diagnosed with CP-CML.

**Fig. 1:**
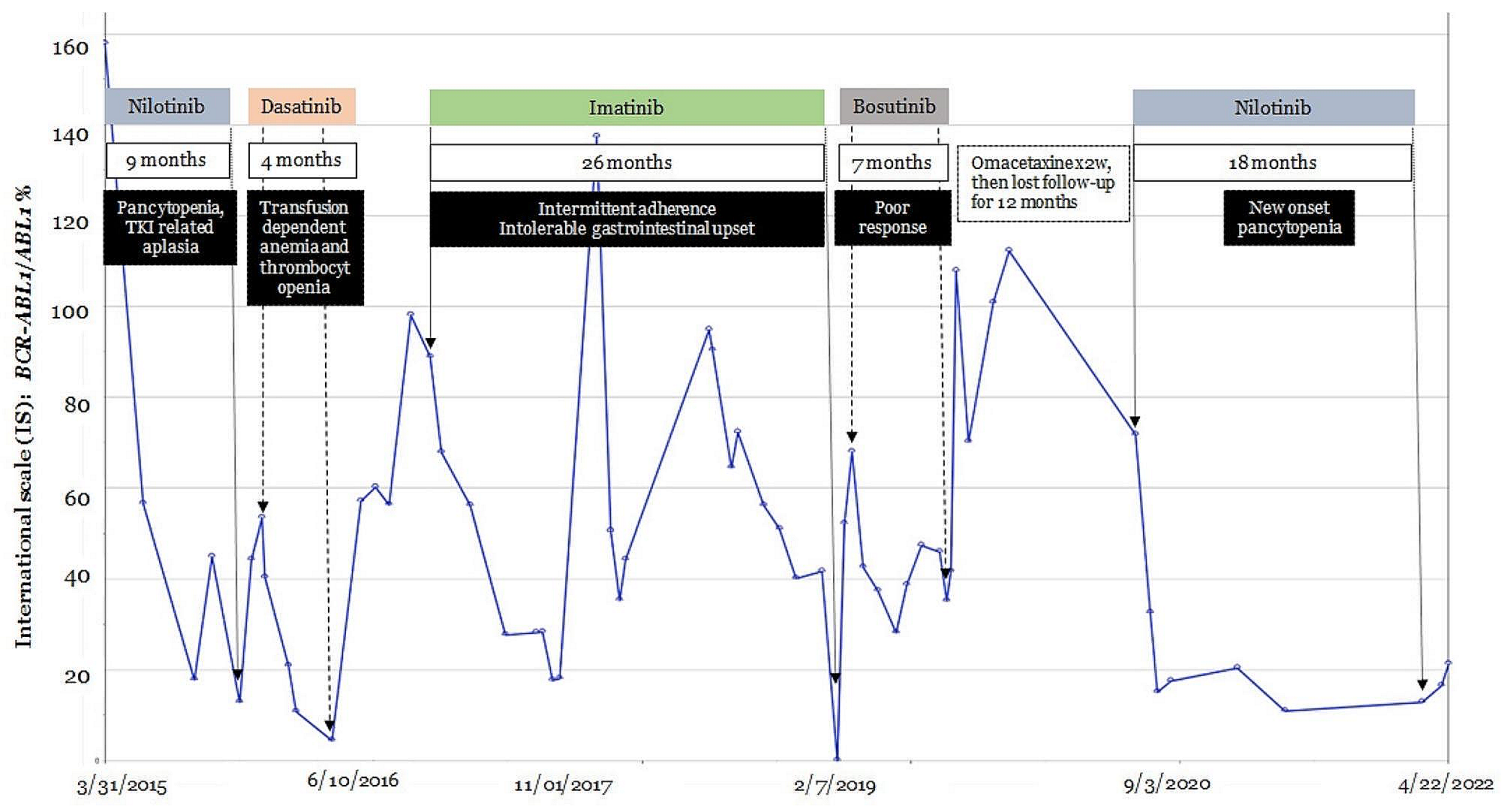
International scale (IS) values (*BCR::ABL1/ABL1*%) are shown over the course of the disease in relation to TKI treatments, including nilotinib, dasatinib, imatinib, and bosutinib, administered from March 2015 to April 2022. The duration of each TKI treatment is indicated in white boxes, while the reasons for discontinuation, such as drug toxicities, intolerance, and non-adherence, are specified in the black boxes. 2w = 2 weeks

Her complex treatment course and molecular responses are depicted in Fig. 1. Initially treated with hydroxyurea for 4 days, she was then started on nilotinib. After 9 months of therapy, *BCR::ABL1* was 12.882% and nilotinib was discontinued due to complete blood count (CBC) showing pancytopenia with WBC of 2.3 × 10^9^/L, absolute neutrophil count (ANC) of 1.6 × 10^9^/L (RR 1.8–7.8 × 10^9^/L), Hgb of 8.8 g/dL, and platelet count of 59 × 10^9^/L (RR 143–382 × 10^9^/L). BM biopsy revealed TKI-related aplasia. *BCR::ABL1* kinase domain mutation analysis was negative. After 2 months off therapy and improvement of her CBC, our patient was started on dasatinib at 100 mg daily. Her *BCR::ABL1* nadired at 4.4% after 4 months, but dasatinib was stopped due to transfusion-dependent anemia and thrombocytopenia. Despite her counts recovering off therapy, her *BCR::ABL1* IS continued to rise, reaching 60–88%. The patient and providers determined that a reduced dose of dasatinib likely would not be sufficient to control the disease, as they were unable to proceed with the full dose. Consequently, she was switched to imatinib 400 mg daily after six months without TKIs. Over the next 26 months, she was intermittently adherent with imatinib. Despite achieving a major molecular response, she was switched to bosutinib given insufferable gastrointestinal upset. She was treated with bosutinib for 7 months before switching to omacetaxine due to inadequate response. Again, mutation analysis was unrevealing. She was evaluated for allogeneic stem cell transplantation (HSCT) and had a fully human leukocyte antigen matched unrelated donor available, but never proceeded forward due to fear. After 2 weeks of omacetaxine therapy, patient suffered a mandibular fracture requiring multiple surgeries. She was lost to follow-up for 1 year but once she reestablished care, she was switched to reduced dose nilotinib for 1.5 years.

In 4/2022, she displayed new-onset pancytopenia (Hb 8.8 g/dL, ANC 1.2 × 10^9^/L, platelets 22 × 10^9^/L), normal WBC 4.2 × 10^9^/L), raising concerns of drug toxicity. Nilotinib was promptly discontinued, and a BM examination was performed.

**Fig. 2:**
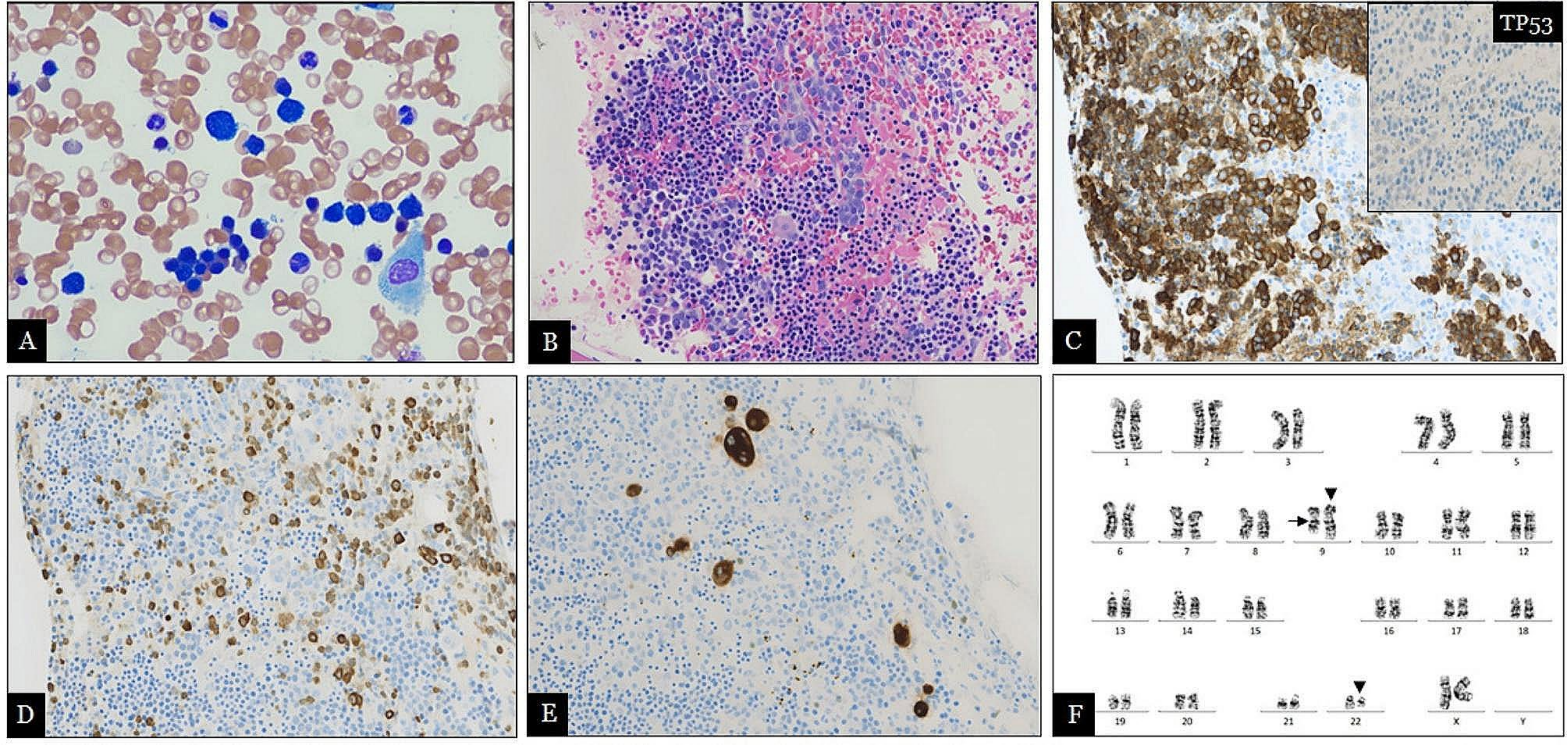
Hemodiluted BM aspirate **(A)** showed predominant erythroid precursors with a full range of maturation without dysplasia and no excess in blasts (Wright Giemsa Stain, original magnification x400). BM core biopsy **(B)** revealed hypercellular marrow with erythroid hyperplasia (90% of cellularity) at various maturing stages, markedly decreased myeloid cells with a reversed M:E ratio of 1:10 (hematoxylin-eosin, original magnification x200). The significantly increased erythroid cells were highlighted by CD71 **(C)**, in which approximately 10% erythroid precursors demonstrated by E-cadherin **(D)**. TP53 is negative (C, inset). CD61 **(E)** showed megakaryocytes with overall unremarkable morphology (original magnification x200). Chromosomal analysis (F) depicted Ph chromosome t(9; 22) detected in 19 out of 20 cells (arrow head), along with inv9 (p12q13) in all metaphase cells, a common chromosomal variant without clinical significance (arrow)

The BM aspirate smears were hemodiluted but showed a predominance of erythroid cells, without dysplasia or an increase in myeloblasts or pronormoblasts (Fig. 2A). Iron staining revealed the presence of storage iron without ringed sideroblasts. The BM core biopsy demonstrated a hypercellular marrow (90%) with a substantial increase in erythroid cells with a reversed M:E ratio of 1:10. Both erythroid and myeloid cells displayed a full range of maturation (Fig. 2B). Megakaryocytes were present in normal numbers with unremarkable morphology. Immunohistochemistry (IHC) analysis highlighted the proliferation of maturing erythroid cells (90% of marrow cellularity) via CD71 staining (Fig. 2C) and erythroid precursors (10% of erythroid cells) via E-cadherin staining (Fig. 2D). TP53 showed a wild-type pattern in erythroid cells (Fig. 2C, inset). CD61 staining highlighted megakaryocytes with variable morphology (Fig. 2E). CD34-positive blasts were not increased, and reticulin staining revealed grade 2/3 reticulin fibrosis (not shown). CML transformation to pure erythroid leukemia (PEL) was ruled out due to the absence of elevated proerythroblasts, the lack of morphological dysplasia, and the absence of TP53 mutation by IHC.

Surprisingly, chromosomal analysis revealed the presence of t(9;22) (q34;q11.2) in 19 out of 20 metaphase cells, along with the previously detected chromosomal polymorphism variant inv9 (p12q13) (Fig. 2F). The IS *BCR:ABL1* level was 20.7%. Comprehensive NGS performed by FoundationOne confirmed the *BCR::ABL1* fusion transcript (p210) without detection of any other pathogenic variants. The diagnosis of the erythroid variant of CML was confirmed based on the disease-defining Ph chromosome.

The patient continues to decline HSCT and is sporadically adherent to a reduced dose of asciminib, currently experiencing transfusion-dependent anemia.

## Discussion and conclusions

We are the first to report the emergence of an erythroid variant of CML in a patient resistant to multiple TKIs. Typically, CML presents with proliferating granulocytes driven by the Ph chromosome. Occasionally, it can manifest atypical features such as significant thrombocytosis or monocytosis, resembling other myeloid neoplasms [11, 12]. BM analysis mirrors these blood features, showing marked granulocytic proliferation resulting an increased M:E ratio.

The presence of predominant erythroid hyperplasia in CML is exceptionally rare, only one such case was reported in 2008, termed the “erythroid variant of CML” by Talreja et al. [3]. This previous case involved a 43-year-old male who displayed a myelodysplastic clinical profile, featuring severe thrombocytopenia (16 × 10^9^/L), mild macrocytic anemia (Hb 10.8 g/dL, mean corpuscular volume 118fL), and normal WBC count (5.6 × 10^9^/L) without monocytosis, neutrophilia, basophilia or eosinophilia. Additionally, splenomegaly was present in this patient. Notably, both our patient and the case reported in 2008 shared similarities, including normal white blood cell counts, mild anemia, and severe thrombocytopenia without characteristic features of CML in the PB. Detailed BM analysis in both cases revealed a hypercellular marrow with erythroid hyperplasia at various stages, harboring the *BCR::ABL*1 (p210) fusion gene. Remarkably, the previously reported patient responded well to imatinib therapy, achieving a major MR.

The blasts in advanced CML are usually of myeloid (granulocytic) or lymphoid lineages [13], with few cases demonstrating erythroid blast crisis [14–16]. PEL transformation from CML is a characterized by a hypercellular marrow dominated by erythroid cells (≥ 90% of marrow cellularity), of which ≥ 30% are proerythroblasts [17]. Erythroid precursors in PEL typically display prominent dysplastic features. Both myeloid and megakaryocytic cells are markedly reduced. PEL cases are commonly associated with biallelic TP53 mutations, and prognosis is poor [18]. Due to overlapping morphologic features, PEL is the primary consideration in the differential diagnosis of the “erythroid variant” of CML. With a careful morphologic examination focusing on dysplasia and erythroblast count, distinguishing between these two entities is not challenging.

**Fig. 3:**
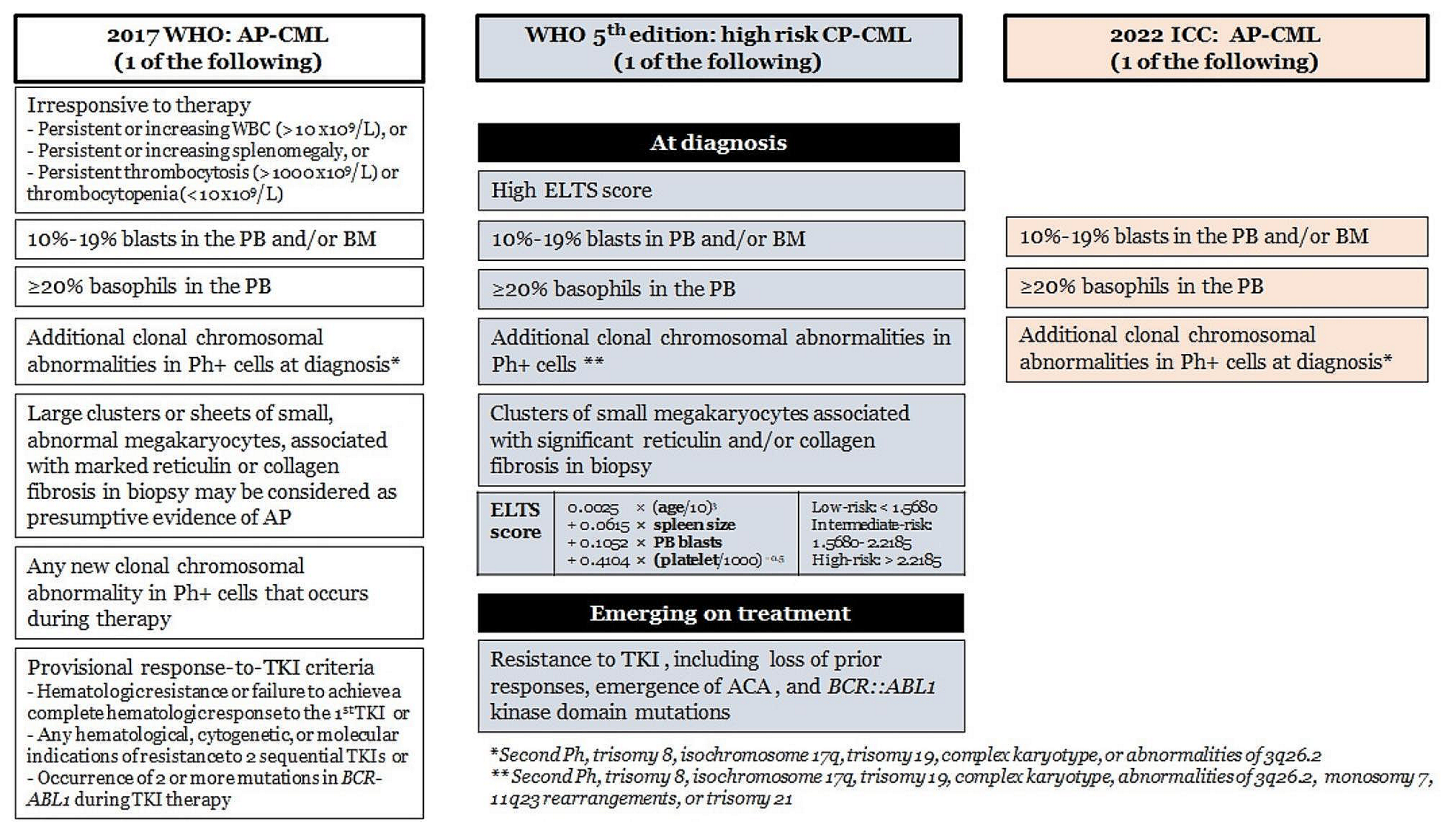
Comparison of diagnostic criteria for “CML-AP” in the 2017 WHO (left row) [17], “high risk CP-CML” according to 5th WHO edition (middle row) [19], and simplified “AP-CML” in the 2022 ICC (right row) [20]. AP = accelerated phase; CP = chronic phase; ELTS = The EUTOS long-term survival score; ACA = additional cytogenetic abnormalities; TKI = tyrosine kinase inhibitor

Recent modifications in the diagnostic guidelines for CML were introduced in 2022 by the World Health Organization (WHO) [19] and the International Consensus Classification (ICC) [20], building upon the previous 2017 WHO classification [17]. In both CP-CML and BP-CML, the diagnostic criteria remained consistent. CP-CML is diagnosed based on the persistence of the *BCR::ABL1* rearrangement, whereas BP-CML requires the presence of ≥ 20% blasts in PB ore BM, myeloid sarcoma, or the existence of lymphoblasts (> 5% as required by ICC, with no specified cut-off by WHO 5th edition). However, notable changes in AP-CML were made by both the 2022 ICC and WHO 5th edition, diverging from the 2017 WHO classification, as depicted in Fig. 3. The WHO 5th edition now eliminates AP-CML and only recognizes only CP-CML and BP-CML. In the era of TKIs, labeling AP-CML has become less practical, and it is now termed as “high-risk CP”, which integrates risk factors for disease progression, such as ETLS score, TKI resistance, additional cytogenetic abnormalities, and *ABL1* kinase mutations (Fig. 3, middle row). The 2022 ICC maintains three phases but notably simplifies the diagnostic criteria for AP-CML to three core criteria (see Fig. 3, right row).

Given these changes, patients may be classified into different disease phases depending on the classification system used. In our unique case, due to resistance to TKI therapy without cytogenetic clonal evolution or *ABL1* kinase domain mutation and the absence of excess blasts and basophilia, the disease falls under the “CP-CML” category according to the 2022 ICC. However, it could also be classified as “AP-CML” according to the 2017 WHO or “high risk CP-CML” per WHO 5th edition. Therefore, it is recommended to report all diagnoses according to both ICC and WHO guidelines, and maintain close communication with the clinical team is essential for a comprehensive understanding of the disease status.

TKIs are the standard therapy for CML and are classified into three generations: first-generation (imatinib), second-generation (dasatinib, nilotinib, and bosutinib), and third generation (ponatinib) [6, 10]. Patients resistant to TKIs might need salvage therapy. Asciminib, which targets the myristoyl pocket of *ABL1*, provides a novel approach for CP-CML patients with resistance or intolerance to previous TKIs, including those with the T315I mutation [10]. Additionally, omacetaxine, an inhibitor of protein synthesis, and HSCT can be considered for advanced CML patients [21].

The progression of CML patients treated with TKIs often involves additional cytogenetic abnormalities [22, 23]. Upon transformation to BP-CML, 70–80% of cases display extra chromosomal abnormalities, including 3q26.2 rearrangement, monosomy 7, trisomy 8, isochromosome 17q, trisomy 19, trisomy 21, additional Ph chromosome, and complex karyotypes [12]. Besides mutations in *ABL1* kinase domain, various somatic mutations, such as *TP53, RUNX1, ASXL1, IKZF1, WT1, TET2, NPM1, IDH1, IDH2, NRAS*, and *KRAS*, have been suggested in the progression from CP-CML to AP/BP-CML, yet there is no identifiable single mutation or specific combination that has been consistently identified [24, 25]. In our case, the absence of cytogenetic progression and pathogenic gene mutations further emphasizes the complexity of CML progression. The underlying mechanism behind this clonal evolution, and how the original neoplastic myeloid cells were suppressed or eliminated, while the neoplastic erythroid subclone gained the growth advantage and evaded TKI treatment, remains unclear.

In summary, this case represents the first report of a CML patient resistant to multiple TKIs who evolved into an erythroid variant. This variant is characterized by a hypercellular marrow with erythroid predominance and the absence of typical CML features. The hallmark for diagnosis lies in the presence of the Ph chromosome within these progressively maturing erythroid cells. Interestingly, our unique case showed no detectable cytogenetic or molecular abnormalities that commonly seen in disease progression. Screening for the Ph chromosome is crucial in patients suspected of having a myeloid neoplasm, even when typical CML features are absent. Correlating with morphology, cytogenetic, and molecular studies, along with engaging in multidisciplinary discussions, is essential for accurate subclassification of CML and for developing the most appropriate treatment plan.

## Data Availability

No datasets were generated or analysed during the current study.

## Abbreviations

ANC: Absolute neutrophil count
AP: Accelerated phase
BM: bone marrow
BP: Blast phase
CML: Chronic myeloid leukemia
CBC: Complete blood count
CP: Chronic phase
CyR: Cytogenetic response
ETLS score: The EUTOS long-term survival score
Hb: Hemoglobin
HSCT: allogeneic stem cell transplant
ICC: the International Consensus Classification
IHC: Immunohistochemistry
M: *E ratio*:Myeloid to erythroid ratio
MR: Molecular response
NGS: Next generation sequencing
PEL: Pure erythroid leukemia
PB: Peripheral blood
Ph chromosome: Philadelphia chromosome
RT-qPCR: Real-time quantitative Polymerase Chain Reaction (PCR)
TKIs: Tyrosine kinase inhibitors
WBC: White blood cell
WHO: The World Health Organization
